# Comparison of Chemical and Electrochemical Approaches to Abacavir Oxidative Stability Testing

**DOI:** 10.3390/s23052776

**Published:** 2023-03-03

**Authors:** Lucie Pražáková, Jan Fischer, Andrew Taylor, Anna Kubíčková

**Affiliations:** 1 Charles University, Faculty of Science, Department of Analytical Chemistry, Albertov 6, 12800 Prague, Czech Republic; 2 FZU—Institute of Physics of the Czech Academy of Sciences, Na Slovance 1999/2, 18221 Prague, Czech Republic

**Keywords:** degradation of pharmaceuticals, abacavir, oxidation, boron-doped diamond electrode, platinum electrode

## Abstract

A novel electrochemical approach using two different electrode materials, platinum and boron-doped diamond (BDD), was employed to study the oxidative stability of the drug abacavir. Abacavir samples were subjected to oxidation and subsequently analysed using chromatography with mass detection. The type and amount of degradation products were evaluated, and results were compared with traditional chemical oxidation using 3% hydrogen peroxide. The effect of pH on the rate of degradation and the formation of degradation products were also investigated. In general, both approaches led to the same two degradation products, identified using mass spectrometry, and characterised by 319.20 and m/z 247.19. Similar results were obtained on a large-surface platinum electrode at a potential of +1.15 V and a BDD disc electrode at +4.0 V. Degradation of 20% of abacavir, the rate required for pharmaceutical stability studies, took only a few minutes compared to hours required for oxidation with hydrogen peroxide. Measurements further showed that electrochemical oxidation in ammonium acetate on both types of electrodes is strongly pHdependent. The fastest oxidation was achieved at pH 9. The pH also affects the composition of the products, which are formed in different proportions depending on the pH of the electrolyte.

## 1. Introduction

Drug stability studies are an essential part of any new drug development process, providing information on changes in the stability of a drug that may compromise patient safety by creating toxic degradation products. In degradation studies, the active pharmaceutical ingredient (API) is subjected to extreme physical and chemical stresses over a very short time via exposure to UV, oxidation, heat, or high/low pH to accelerate degradation [1,2,3]. According to the International Council for Harmonisation of Technical Requirements for Pharmaceuticals for Human Use (ICH), in general, drugs should be subjected to oxidative stress as part of a forced degradation study [4]. This oxidation is accomplished via electron transfer using addition of a 0.1–3% hydrogen peroxide solution to the drug or API at both ambient and elevated temperatures for 1–7 days [5]. The exact conditions depend on the susceptibility of the drug to undergo oxidation. Oxidation by hydrogen peroxide should mimic conditions and degradation products that can be formed as impurities during long-term stability studies [6,7]. The level of degradation must be controlled to avoid excessive sample decomposition. This may lead to the formation of secondary degradants, which would not be observed in long-term storage stability studies. The recommended extent loss of API, due to degradation, is usually 5–20% [8,9]. The type and amount of the degradation products are most often monitored by high performance liquid chromatography with mass detection (HPLC/MS) [10].

Electrochemical degradation as a method for decomposing a wide range of electrochemically active compounds has been reported in several works. In these works, the aim was complete decomposition of environmental pollutants including pharmaceuticals [11,12,13,14]. With these findings, we know that partial electrolysis can be applied to pharmaceutical stability studies [15,16,17]. Electrochemical degradation is reported to be faster and to provide the same degradation products as using hydrogen peroxide [18]. The advantages of electrochemical oxidation are mainly the acceleration of stress studies, a more accurate prediction of the degradation of API, and a characterization of degradation products. In addition, low consumption of test substances, low acquisition, and operating costs are also advantages of the method. Electrochemical approaches have already been successfully employed for stress testing of canagliflozin and fesoterodine in a flow-through electrochemical cell [19,20]. 

A key parameter in the design of systems for electrochemical degradation is the electrode material, which may consist of Pb/PbO_2_, various modified glassy carbons, platinum, and next-generation electrodes, such as BDD [21,22,23,24]. BDD is a widely used electrode for oxidisation of non-electrochemical active organic compounds through its generation of OH radicals. Compared to platinum, BDD electrodes have a wide potential window and are more resistant to passivation of the electrode surface. Organic compounds are oxidised by BDD electrodes via the direct transfer of electrons from the BDD surface to the compound at higher potentials, as well as indirect oxidation by hydroxyl radicals generated on the electrode surface. The indirect oxidation route allows the oxidation of organic compounds at higher positive potentials than other electrode materials [12,25]. 

**Figure 1:**
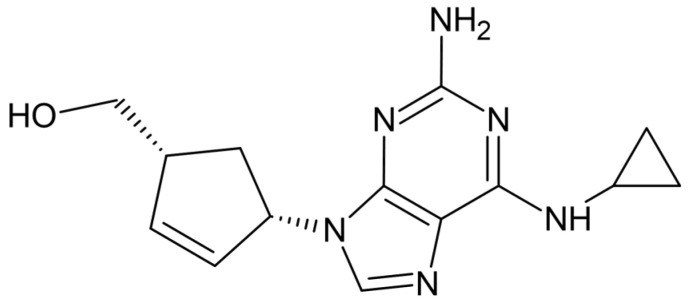
Chemical structure of abacavir.

This study works with abacavir (((1*S*,4*R*)-4-(2-amino-6-(cyclopropylamino) purin-9-yl) cyclopent-2-en-1-yl) methanol, (for its structure, see Figure 1) as a model substance. Abacavir is the API of antiretroviral drugs used against the human immunodeficiency virus (HIV). Abacavir belongs to a class of drugs called nucleoside reverse transcriptase inhibitors as its mechanism of action is analogous to a carbocyclic 2′-deoxyguanosine nucleoside. The nucleoside analogue competitively inhibits HIV reverse transcriptase and terminates the elongation of the proviral DNA chain and reduces the amount of HIV in the blood [26,27].

Degradation studies on abacavir have been carried out predominantly using hydrogen peroxide oxidation by Vukkum et al. [28], Rao et al. [29], Kurmi et al. [30], and Prakash et al. [31]. The experimental conditions vary from study to study (for details see Table 1). Therefore, the resulting degradation products from these studies do not match each other. The most frequently mentioned degradation products are products with m/z 303.20 [28,29,30], 319.20 [28,31], and 247.19 [29,30,31]. Other products are also briefly mentioned. For a detailed list of the products cited in the literature and their m/z, see Table S1 in Supplementary Materials.

The only electrochemical study of abacavir oxidation has been provided by Zhou et al. [22]. In this case, the purpose of the study was the complete degradation of abacavir, and the determination of its toxicity directed to aquatic animals using the penetration current of a porous Ti/SnO_2_—Sb anode. The electrochemical degradation of abacavir is reported to be faster and to provide the same degradation products as using hydrogen peroxide. Two degradation products were identified with m/z 319.20 and 247.19. The voltammetric behaviour and mechanism of the electrode reactions of abacavir were previously reported on a glassy carbon disk electrode [32].

The aim of this study is to develop and deepen the capabilities of electrochemical oxidation of pharmaceuticals and compare established methods (chemical oxidation with hydrogen peroxide [33]) with novel electrochemical approaches. The oxidation process was studied using a glass batch cell with a conventional platinum electrode and a BDD electrode, which allows fine-tuning of oxidation conditions for drug stability testing.

## 2. Materials and Methods

### 2.1. Chemicals

Chemicals used in this study included: Abacavir Sulfate (CAS 188062-50-2) ≥ 99.0% purity (Sigma-Aldrich, Zwijndrecht, The Netherlands), Acetonitrile LC-MS grade (Honeywell, Offenbach am Main, Germany), ammonium acetate purity grade ≥ 98.0% (Sigma-Aldrich, The Netherlands), acetic acid 100% purity for LC-MS (Merck, Darmstadt, Germany), ammonium hydroxide 25% p.a. purity (Lach-Ner s.r.o., Neratovice, Czech Republic), hydrogen peroxide 30% p.a. purity (Lach-Ner s.r.o., Czech Republic), silver nitrate purity ≥ 99.0% (Lach-Ner s.r.o., Czech Republic), and sulphuric acid purity 96% (Penta, Prague, Czech Republic). Water was used for UHPLC/MS (Honeywell, Germany). Water for experiments was obtained by purifying demineralized water using an ELGA purification instrument (Metrohm, Herisau, Switzerland).

### 2.2. Instruments

A Waters Acquity UPLC H Class (Waters, USA) with a high-pressure pump, autosampler, thermostat, PDA detector controlled by Empower3 software (Waters, Milford, MA, USA), and mass spectrometer with mono quadrupole QDa (Waters, USA) was used for liquid chromatography analyses. Cyclic voltammetry, with a scan rate of 100 mV s^−1^, and a three-electrode electrochemical cell for electrochemical oxidation up to +2.50 V was performed on an Eco-Tribo Polarograph potentiostat (Polaro-Sensors, Prague, Czech Republic) with Multielchem software (version 3.2.1). Electrodes used in potentiostatic oxidation were: a working BDD circular electrode (0.20 cm^2^) with a B/C ratio of 4000 ppm (FZU—Institute of Physics of the Czech Academy of Sciences, Prague, Czech Republic), an auxiliary platinum counter electrode (Electrochemical Detectors, Czech Republic), and a referent electrode Ag|AgCl|3.00 mol L^−1^ KCl (Electrochemical detectors, Brno, Czech Republic). An Owon P4603 power supply (Owon, Zhangzhou, China) was used for electrochemical oxidation above +2.50 V using the same working BDD electrode and platinum counter electrode. Electrolysis was performed with a potentiostat/galvanostat model 273 EG&G (Princeton applied research, Oak Ridge, TN, USA) with a large-area platinum mesh (geometric area 53 cm^2^) working electrode; another platinum mesh was used as an auxiliary electrode, and the reference electrode was silver in 0.1 mol L^−1^ AgNO_3_. Determination of pH was performed using a Jenway 3540 pH meter/conductometer (Bibby Scientific, Staffordshire, UK) and the use of calibration buffers (pH = 4.0, 7.0, 10.0, Merck, Germany). For sonification of samples, an Elmasonic S15H ultrasonic bath (Elma, Singen, Germany) was used.

### 2.3. Chemical Oxidation

The standard procedure for chemical oxidation of abacavir was as follows: Working solutions were prepared for degradation studies with abacavir at a concentration of 1 mmol L^−1^ (0.335 mg mL^−1^). Abacavir was dissolved in 3% (*v*/*v*) hydrogen peroxide, which was prepared by diluting 30% hydrogen peroxide. Abacavir solutions were placed in a degradation chamber and subjected to oxidation at 25 and 50 °C for 1, 2, 3, 5, and 24 h. 

### 2.4. Electrochemical Oxidation

An ammonium acetate buffer solution (0.20 mol L^−1^) was prepared by weighing the required amount of ammonium acetate and then dissolving in deionized water. The pH of the ammonium acetate solutions was adjusted to pH = 4.0, 7.0, and 9.0 using acetic acid and 25% ammonium hydroxide. Subsequently, the required amount of abacavir sulfate was weighed and dissolved in ammonium acetate solutions (0.15; 0.50 mmol L^−1^) with appropriate pH. Prepared solutions were filtered through a Nylon 66 Membranes (Supelco) filter with a pore size of 0.20 μm using a vacuum pump and then sonicated for 30 min. A solution of 0.01 mol L^−1^ silver nitrate and 0.20 mol L^−1^ ammonium acetate was prepared by weighing solid silver nitrate and ammonium acetate and dissolving in deionized water. The solution was sonicated for 15 min after the addition of deionized water.

#### 2.4.1. Apparatus with Platinum Working Electrode

The working solution of abacavir was electrochemically oxidised in a three-electrode apparatus consisting of a potentiostat with a large-volume glass batch cell (25 mL) with a platinum mesh working electrode. A second platinum mesh immersed in 40 mL of ammonium acetate solution (0.20 mol L^−1^) was used as an auxiliary electrode. A silver electrode immersed in 10 mL of a solution of silver nitrate (0.01 mol L^−1^) and ammonium acetate at a concentration of 0.20 mol L^−1^ was used as reference electrode. All electrode compartments were separated by a salt bridge filled with ammonium acetate solution (0.20 mol L^−1^). Platinum electrodes were cleaned before each measurement via annealing in an upper reducing flame of a flame torch for 3 min. The analyte solution was stirred using a magnetic stirrer. For ultra-high performance liquid chromatography with mass detection (UHPLC/MS) analysis, 200 μL of oxidised solution was collected.

#### 2.4.2. Apparatus with BDD Working Electrode

A apparatus for electrochemical degradation of abacavir was assembled in two ways. Both apparatuses consisted of 1 mL volume glass batch cells without separate electrode compartments. Electrochemistry oxidation at a degradation potential up to +2.50 V and voltammetric measurements were carried out using three electrode apparatuses with a potentiostat. For degradation potential above +2.50 V, two electrode arrangements with a power supply were used. A BDD electrode and a platinum electrode were used as working and auxiliary electrodes, respectively. In both apparatuses, the analysed solutions were stirred by a glass stirrer. After oxidation, 200 μL of oxidised solution was taken for UHPLC/MS analysis. The BDD electrode was anodically cleaned using sulfuric acid (c = 0.50 mol L^−1^) for 10 min at a constant potential for three electrode apparatuses +2.5 V and for two electrode apparatuses +4.5 V before each measurement.

### 2.5. UHPLC/MS Method

Measurements were performed on a Waters Acquity UPLC H Class with a Kinetex C18 column (100 × 2.1 mm, 1.7 μm, Phenomenex, Torrance, CA, USA). The mobile phase consisted of acetonitrile (A) and ammonium acetate (c = 20 mmol L^−1^, pH = 7.0 (B)). The gradient program was set as follows: *t* (min)/%(*v*/*v*) A: 0/10; 2/10; 7/ 70; 8/70; 9/10; 11/10. The flow rate was 0.3 mL min^−1^, the injection volume was 1 μL, the column temperature was 28 °C, and the temperature of the autosampler was maintained at 10 °C. The total analysis time was 11 min. Samples were analysed using a PDA detector at a wavelength of λ = 254 nm.

The QDa mass spectrometer worked in TIC mode. Ionisation took place in positive mode, mass range 100.00–500.00 Da, cone voltage 15 V, probe temperature 600 °C, and positive and negative capillary voltage 0.8 kV. Ions were recorded over 1–7 min.

A ammonium acetate mobile phase (c = 20 mmol L^−1^) solutions were sonicated for 15 min and filtered through a Nylon 66 Membranes filter (Supelco) with a pore size of 0.20 μm using a vacuum pump.

## 3. Results and Discussion

### 3.1. Chemical Oxidation

**Figure 2:**
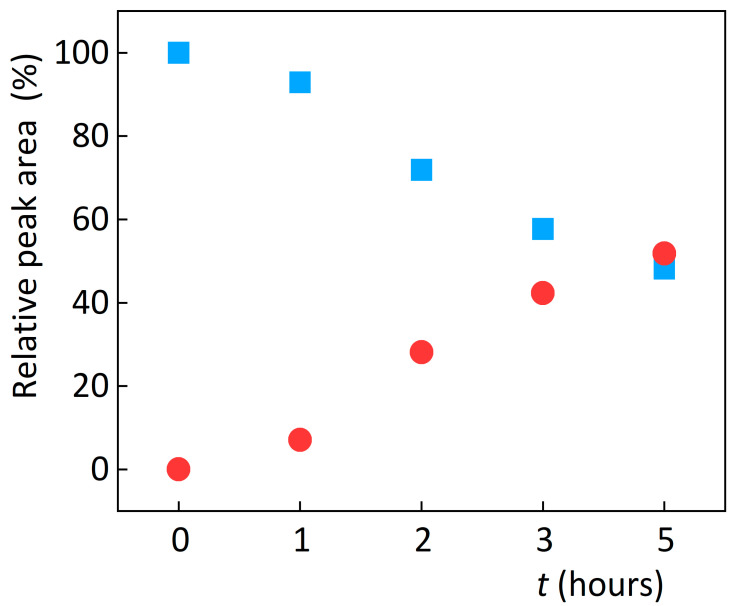
Effect of degradation time on relative peak area of abacavir (squares) and its degradation product OP1 (circles) during chemical oxidation using hydrogen peroxide.

According to commonly used recommendations [5,8,9], abacavir was subjected to peroxide-mediated oxidation stress with 3% hydrogen peroxide at 25 and 50 °C to compare the extent of degradation and the resulting products of this commonly used degradation procedure with electrochemical degradation. The degradation rate and resulting oxidation products were evaluated using UHPLC/MS. Degradation at 25 °C took 24 h to reach the desired 15% degradation. Therefore, to accelerate the process, degradation was carried out at 50 °C, during which time the desired 15% drop in the amount of abacavir occurred in less than two hours. Three degradation products were formed during oxidation, only one of which is considered the main oxidative degradation product OP1 characterised by a relative retention time (RRT) of 0.40 and an m/z of 319.20. The other two with RRT 0.30 and 0.45 are below the level for quantification as degradation products. The formation of this degradation product is consistent with the results published in studies [22,28,29,30]. Figure 2 shows degradation development expressed as a decrease in abacavir-relative peak area and simultaneous formation of the degradation product OP1. 

### 3.2. Electrochemical Oxidation

Since oxidation with hydrogen peroxide takes more than an hour even at elevated temperatures, it was decided to test oxidative degradation using electrochemical principles. The advantages of electrochemical oxidation over hydrogen peroxide oxidation are its speed, low sample consumption, and miniaturisation of the process, which reduces the cost of materials and the amount of API used. Another major advantage is the more realistic simulation of oxidation as an external oxidising agent is not introduced into the sample. Moreover, chemical oxidation with hydrogen peroxide at higher temperatures can cause rapid oxidative decomposition of the sample and the formation of oxidation products, which are unlikely to occur during drug storage [34].

Electrochemical oxidation was performed using two different electrode materials and the effectiveness on degradation was assessed. A platinum electrode was chosen as a representative of the classic electrode material for oxidation in the potential window, and a BDD electrode was chosen as a new promising material providing efficient degradation, even beyond the potential window of the electrode. Degradation was carried out in batch cells, which can be easily modified, and the volume of the solution was stable. For degradation on a platinum working electrode, a classic electrochemical cell with separate electrode compartments using salt bridges was used. Subsequently, a more user-friendly low-volume cell without a separate auxiliary electrode was used for BDD investigation.

**Figure 3:**
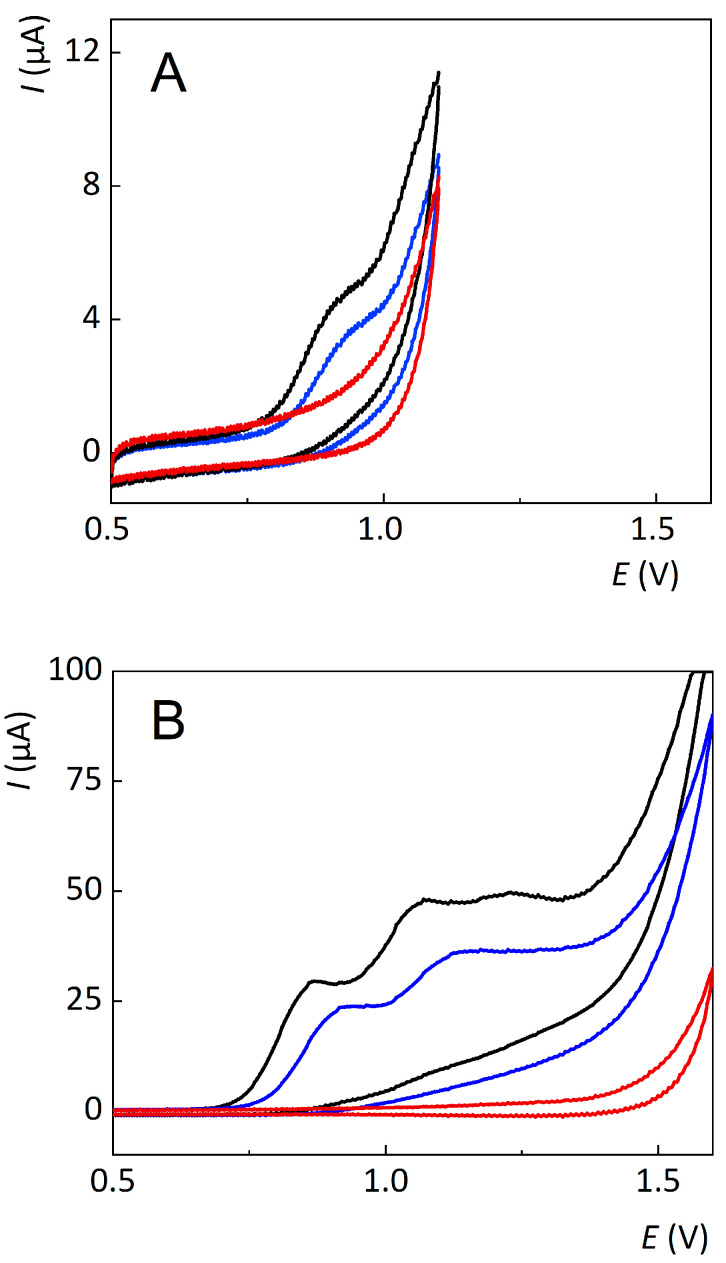
Cyclic voltammograms of abacavir solution in ammonium acetate (c = 0.20 mol L^−1^) at pH = 7.0 (black line is the first scan, blue line is the second scan) and the basic electrolyte (red line) on platinum (**A**) and BDD electrodes (**B**).

The electrochemical properties of abacavir and the range of potential windows on the platinum and BDD working electrodes were investigated using cyclic voltammetry in an ammonium acetate (c = 0.20 mol L^−1^)- supporting electrolyte. On a platinum electrode, abacavir is oxidised giving one voltammetric wave in the range of +0.80 to +1.00 V, as shown in Figure 3A. The voltammetric wave corresponds to the previously described mechanism [32], but the measurement is limited by the edge of the potential window on platinum at +1.10 V. Therefore, for platinum electrodes, a maximum oxidation potential of +1.15 V was used for the electrochemical degradation of abacavir. At higher potentials, decomposition of the supporting electrolyte occurred, and thus erroneous degradation products could be produced. The determination of the most efficient degradation potential in a potential window of +0.30 to +1.15 V was investigated for each potential in increments of 0.10 V. Abacavir degraded more with increasing potential and reached the maximum degradation at +1.15 V. On a BDD electrode, abacavir is oxidised in three voltammetric waves at the wave peak potential at +0.85, +1.05, and +1.25 V, as shown in Figure 3B. The first oxidation wave is the same for both electrode materials, and the two extra waves from the BDD electrode cannot be observed on the platinum electrode due to its narrow potential window. With every additional scan cycle, the oxidation potential of the waves increased. This indicates electrode passivation and therefore that the electrode must be anodically activated before each new measurement.

Abacavir shows a similarity in oxidation to adenine [35]. Compared to adenine, abacavir has only one carbon, which is able to form a carbonyl group due to substitutions in the rings. Most likely, only hydrolysis can be realised. The second peak corresponds to oxidation of adenine dimers. The third signal was observed in the literature only at higher concentrations [33], which is also located beyond the potential window of electrodes such as platinum.

**Figure 4:**
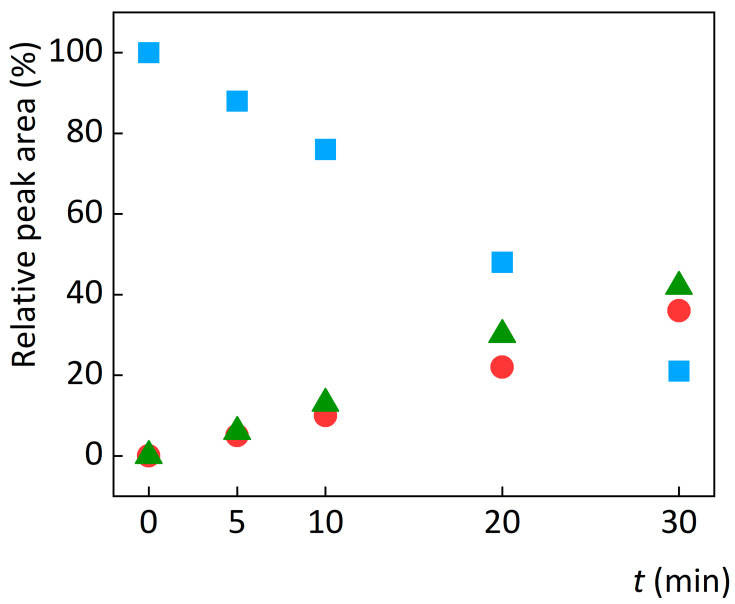
Effect of degradation time on the relative peak area of abacavir (squares) and its degradation products OP1 (circles) and OP2 (triangles) during electrochemical oxidation with a platinum electrode.

Oxidation of abacavir on the platinum electrode to the desired degradation value of 15% took only 7 min. Two oxidation products with RRT = 0.40 and 0.45 were formed and identified using UHPLC/MS. The first oxidation product (OP1) had m/z of 319.20, which corresponds to one of the oxidation products described in several works [18,28,31]. The second oxidation product (OP2) had m/z of 246.90, which corresponds to the product described in [18,29,30,31]. The amount of abacavir (expressed as the relative peak area of abacavir) gradually decreased while the amount of the two oxidation products increased with time (see Figure 4). The oxidation products, OP1 and OP2, were produced almost in the same proportion throughout the oxidation process. The repeatability of the oxidation process was evaluated using relative standard deviation (RSD) of five consecutive oxidations. The RSD value of the abacavir relative peak area was 3.5%.

From the presence of the amine group of abacavir (pKa = 5.80, calculated with [32]) (see Figure 1), it is evident that the pH value can affect the dissociation of abacavir and further influence its degradation. Electrochemical degradation of abacavir was monitored in ammonium acetate at three different pH levels = 4.0, 7.0, and 9.0 at room temperature. At all pH values, the same products were formed, namely OP1 with an RRT of 0.40 and OP2 with an RRT of 0.45. The fundamental difference, however, is in the rate of degradation. The results show that abacavir was oxidised from about 15% in ammonium acetate at pH = 4.0 in 10 min, at pH = 7.0 in 6–7 min, and at pH = 9.0 in 4–5 min. Clearly, abacavir oxidised the quickest at pH = 9.0. Degradation at different pH resulted in different ratios of degradation products. At pH = 9.0, where abacavir degraded the fastest, OP1 was dominantly formed. At pH = 7.0, almost identical amounts of both products were formed. At pH = 4.0, the state reversed, and OP2 was preferably formed. 

The other electrode material that was tested for abacavir degradation was a boron-doped diamond (BDD) electrode. Compared to platinum electrodes, BDD electrodes have a larger potential window, are more resistant to passivation of the electrode surface. Organic compounds can be oxidised at BDD in an indirect way, and oxidation can occur via hydroxyl radicals generated on the electrode surface. The indirect oxidation mechanism also allows the oxidation of organic compounds outside the potential window [36].

For BDD electrodes, based on the experience of the oxidation potentials of abacavir on platinum electrodes, the effect of increasing the potential from +0.40 V to beyond the edge of the potential window into the radical region to +4.50 V was studied. Oxidation provided the same two major degradation products, OP1 and OP2, as observed for the platinum electrode oxidation. At potentials higher than +2.00 V, oxidation by hydroxyl radicals occurred and therefore, production of different degradation products was possible. However, according to UHPLC/MS analysis, the same degradation products are formed in the oxidation using direct electrochemical reaction of abacavir with BDD electrodes as platinum electrodes and hydrogen peroxide. The effect of potentials greater than +2.00 V on the degradation of the abacavir was noticeably greater than at lower voltage. Optimal degradation of abacavir up to the chosen 15% was achieved after 10 min at a voltage of +3.00 V. For comparison, the fastest degradation was observed at a voltage of +4.00 V, when abacavir degraded to 43% after 10 min. At a voltage of +4.50 V, further reactions can occur on the electrode surface and therefore the degradation efficiency decreased. Degradation on a BDD electrode can occur faster than on a platinum electrode at high voltage. The same degradation products were formed on both electrode materials, and the product ratio is comparable. The repeatability of the oxidation process was determined from the five consecutive oxidations and evaluated as the relative value (RSD). RSD values of the peak areas of the abacavir and both degradation products OP1 and OP2 were around 5%.

**Figure 5:**
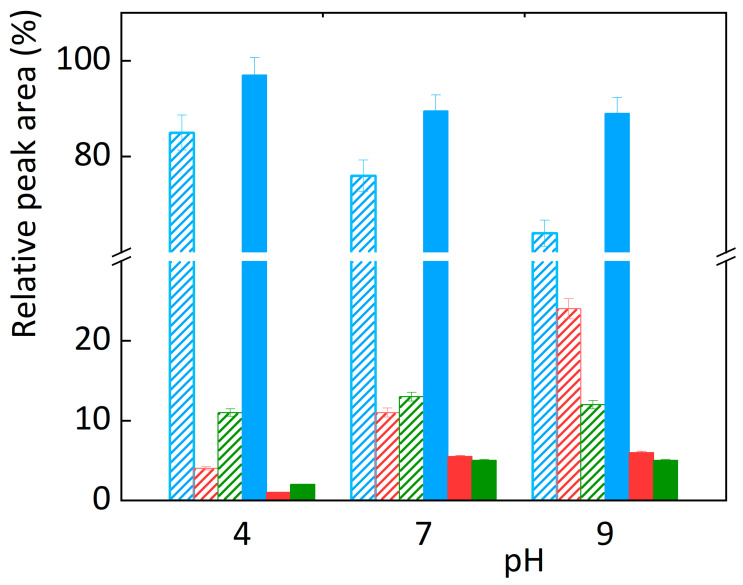
Abacavir degradation level as a function of pH and formation of oxidation products OP1 (red colour) and OP2 (green colour) using platinum (line pattern) and BDD (full colour) electrodes for 10 min at pH = 4.0, 7.0, and 9.0.

As observed on the platinum electrode, the effect of pH on oxidation was studied using the BDD electrode in ammonium acetate solution with pH = 4.0, 7.0, and 9.0 (see Figure 5). Two major degradation products, OP1 and OP2, were again formed. The degradation of abacavir was significantly faster at pH = 7.0 and 9.0 at a potential of 3.00 V. At lower potentials, the degradation rate was negligibly affected by pH compared to degradation at +3.0 V. At a voltage of +3.0 V and pH = 7.0 and 9.0, abacavir degradation occurred at the fastest rate. The reason for the lower rate of oxidation in an acidic environment is probably related to the pKa = 5.8 of abacavir and the change in the mechanism of degradation in protonated and deprotonated forms. When oxidation was performed using direct electrochemical reaction of abacavir directly on the surface of BDD electrodes, i.e., within the potential window, the change in pH had almost no effect. Therefore, the change in pH of the solution does not affect the degradation of abacavir and the formation of different degradation products with chemical structures, but the amount and ratio of degradation products formed on both electrodes.

### 3.3. Comparison of Electrochemical and Chemical Oxidation Methods

**Figure 6:**
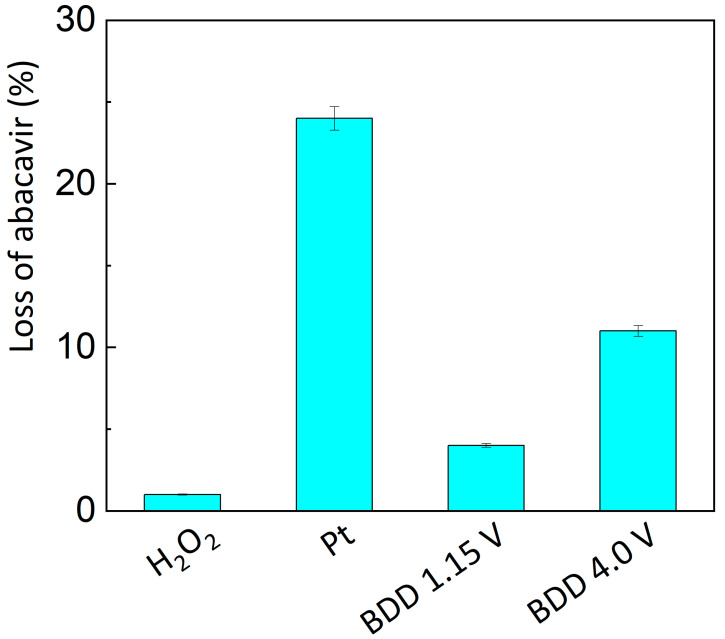
Comparison of the amount of abacavir degraded within 10 min by different methods. Conditions: Chemical oxidation using 3% hydrogen peroxide at 50 °C, and all the electrochemical oxidations occurred at pH 9.

When comparing electrochemical to chemical degradation with hydrogen peroxide (see Figure 6), the most significant difference is in the speed of abacavir degradation. While the degradation by hydrogen peroxide oxidation even at elevated temperatures needs more than 1 h to complete, electrochemical oxidation provides a reasonable level of degradation of abacavir in 10 min. When comparing the efficiency of the electrochemical degradation, it should be considered that the ratio of the electrode area to the volume of the analysed solution was more than six times more favourable for the platinum electrode. Therefore, the results achieved on the platinum and BDD electrodes at +1.15 V are comparable. During chemical degradation, only one degradation product (OP1) is formed, whereas two products (OP1, OP2) were formed by electrochemical degradation. The formation of a larger amount of OP2 may be influenced by the different mechanisms of the subsequent degradation of the intruding products. At the electrodes, the reaction takes place only in the vicinity of the working electrode, whereas in chemical degradation the reaction takes place in the whole volume. During electrochemical degradation, the rate of degradation was almost comparable and was influenced by pH. The fastest degradation was achieved at pH = 9.0. The advantage of degradation on the BDD electrode is the possibility of degradation in the radical region, which is very fast. Analysis by MS spectra confirmed identical impurities in both oxidation methods. Both degradation products, OP1 and OP2, have been reported in the literature [18,28,29,30,31]. Based on the data obtained, the greatest advantage of electrochemical oxidation appears to be the acceleration of stress studies. Furthermore, since electrochemical oxidation produces more degradation products than peroxide-mediated oxidation, API degradation can be more accurately predicted and subsequently characterised. In the future, this method could replace commonly used chemical oxidative procedures in documentation submitted by drug companies when applying for market authorization.

## 4. Conclusions

Electrochemical oxidation on BDD and platinum working electrodes proved to be an effective tool for studying oxidative degradation of pharmaceuticals. Two degradation products, OP1 (319.20) and OP2 (m/z of 246.90), were observed. Degradation products formed by electrochemical oxidation are consistent with those reported in the literature obtained using standard hydrogen peroxide oxidation. The main advantage lies in the time saved when using the electrochemical approach; degradation of 20% abacavir is completed in less than 10 min. Electrochemical oxidation has been shown to be pH dependent. Oxidative decomposition is fastest at pH 9. In addition to pH, the working potential must be chosen carefully. +1.15 V was evaluated as the optimal potential for oxidation with the platinum electrode, whereas the oxidation on BDD took place the quickest at +4.00 V. Finally, it has been demonstrated that the electrochemical approach has the potential for studying oxidative degradation of many other pharmaceutical substances.

## Figures and Tables

**Table 1:** Overview of published methods on abacavir oxidation.

Author	Method of Oxidation	Temperature	Time of Oxidation	Reference
Vukkum et al.	3% H_2_O_2_	Laboratory	7 days	[28]
Rao et al.	6% H_2_O_2_	Laboratory	7 days	[29]
Kurmi et al.	15% H_2_O_2_	30 °C	10 days	[30]
Prakash et al.	30% H_2_O_2_	80 °C	30 min	[31]
Zhou et al.	0.2 mA cm^−2^, Ti/SnO_2_-Sb anode	Laboratory	10 min	[18]

## Data Availability

Data is contained within the article.

## Supplementary Materials

The following supporting information can be downloaded at: https://www.mdpi.com/article/10.3390/s23052776/s1. Table S1: Summary table of abacavir oxidation products formed during degradation studies.
