# Supplementary material for: Comparison of Chemical and Electrochemical Approaches to Abacavir Oxidative Stability Testing
Source: Sensors (Basel). 2023 Mar 3;23(5):2776. doi: 10.3390/s23052776 (PMC10007081; doi:10.3390/s23052776)
Supplement: Supplementary file 1 [file sensors-23-02776-s001.zip › sensors-2219443-supplementary.pdf]

Supplementary Materials

L. Pražáková, J. Fischer, A. Taylor, A. Kubíčková: **Comparison of chemical and electrochemical approaches to abacavir oxidative stability testing**

Table S1. Summary table of abacavir oxidation products formed during degradation studies.

Author	Oxidation product	*m/z*	Expected formula	Reference
Vukkum et al.	OX1	191.10	-	[28]
	OX2	303.20	$C_{14}H_{19}N_6O_2^+$	
	OX3	223.20	-	
	OX4	319.20	$C_{14}H_{19}N_6O_3^+$	
Rao et al.	OX2	303.20	$C_{14}H_{19}N_6O_2^+$	[29]
	OX5	247.19	$C_{11}H_{15}N_6O^+$	
Kurmi et al.	OX2	303.20	$C_{14}H_{19}N_6O_2^+$	[30]
	OX5	247.19	$C_{11}H_{15}N_6O^+$	
	OX6	321.17	$C_{14}H_{21}N_6O_3^+$	
	OX7	303.15	$C_{14}H_{19}N_6O_2^+$	
Prakash et al.	OX4	319.22	$C_{14}H_{19}N_6O_3^+$	[31]
	OX5	247.19	$C_{11}H_{15}N_6O^+$	
Zhou et al.	OX5	246.90	$C_{11}H_{15}N_6O^+$	[18]
	OX4	319.20	$C_{14}H_{19}N_6O_3^+$	
	OX8	151.07	$C_5H_7N_6^+$	
